# Optimisation of the Protocol for the LIVE/DEAD^®^ BacLight^TM^ Bacterial Viability Kit for Rapid Determination of Bacterial Load

**DOI:** 10.3389/fmicb.2019.00801

**Published:** 2019-04-12

**Authors:** Julia Robertson, Cushla McGoverin, Frédérique Vanholsbeeck, Simon Swift

**Affiliations:** ^1^Department of Molecular Medicine and Pathology, The University of Auckland, Auckland, New Zealand; ^2^The Dodd-Walls Centre for Photonic and Quantum Technologies, Auckland, New Zealand; ^3^Department of Physics, The University of Auckland, Auckland, New Zealand

**Keywords:** viability, fluorescence, *Escherichia coli*, SYTO 9, propidium iodide, crosstalk

## Abstract

Rapid antimicrobial susceptibility testing is needed to reduce prescription of inappropriate antibiotics. A rapid alternative to standard culture-based testing is to determine reductions in cell viability using the LIVE/DEAD^®^ BacLight^TM^ Bacterial Viability Kit. We optimised the kit protocol for this application, focusing on simplifying the process by minimising the steps involved and on determining the optimal analytical parameters for fluorescence measurements from the dyes SYTO 9 and propidium iodide (PI). We demonstrate that for our experimental system, the intensity of emissions should be integrated from 505–515 nm for SYTO 9 and 600–610 nm for PI, and the proportion of live cells calculated from a new dye ratio formula, termed the adjusted dye ratio. We show that the pre-staining washing step is not necessary if a non-fluorescent growth media is used; however, staining must be done for each sampling as prolonged exposure to the dyes negatively impacts cell viability. The optimised methodology was able to reproducibly detect reductions in culture viability when the proportion of live cells in a sample of 1 × 10^8^ cells/ml fell below ∼50% live in a media that supports the growth required for detecting antibiotic killing. Finally, we show that the interaction of fluorescence emission spectra from SYTO 9 and PI stained *Escherichia coli* cells is influenced by the proportion of dead cells in a sample. The excitation of PI by SYTO 9 was found to occur in populations containing sufficient numbers of dead cells (>25%), whereas in populations with low numbers of dead cells the dye interaction was additive in regard to red emissions, indicating that these dye interactions may offer another dimension to live/dead analysis. Fluorescence measurements from samples established according to the optimised protocol can be taken using a flow cytometer, spectrofluorometer, microplate reader, and the Optrode, a fibre-based spectroscopic system developed at the University of Auckland.

## Introduction

Rates of antimicrobial resistance (AMR) are an increasingly significant health burden, particularly in bacteria that cause common infections (WHO, 2014, 2017). A key strategy to address the problem of AMR is improved infection control, of which fast and reliable diagnostics is an important aspect.

Antimicrobial susceptibility testing (AST) is used to determine the sensitivity of bacteria to potential antibiotic treatments (Jorgensen and Ferraro, 2009). Culture-based techniques are typically used, where live cells grow on bacteriological media while dead cells do not (Berney et al., 2007; Jorgensen and Ferraro, 2009). Culture-based methods only give information on viable and culturable cells, and are prone to errors due to factors involved in bacterial growth on agar plates (Davis, 2014). Further, culture-based methods are slow [typically obtained in two to five days (Lagier et al., 2015)], which can lead to prescription of an inappropriate antibiotic in a clinical setting due to urgency in treatment (WHO, 2014). Use of the wrong antibiotic leads to treatment failure, increased patient morbidity and mortality, and can promote the development of further AMR in bacteria (WHO, 2014, 2017). Improved, faster diagnostics that can inform antibiotic choice may circumvent this problem.

Rapid nucleic acid amplification and mass spectrometry methods have been applied to AST (Lagier et al., 2015; Sparbier et al., 2016). However, the former can only inform about the presence of resistance in a limited number of bacterial species (Sparbier et al., 2016) and the latter about which drugs are not appropriate to use while providing no guidance on selection of an effective antibiotic for treatment (van Belkum et al., 2017). Therefore, despite development of newer methods, there is still a need for faster and more informative AST techniques. An ideal AST method will generate an answer rapidly with minimal processing steps (Jorgensen and Ferraro, 2009). In addition to improved patient care, a rapid AST will decrease costs through reductions in additional laboratory tests, invasive procedures, and durations of hospitalisation (Doern et al., 1994; Barenfanger et al., 1999).

An alternative to culture-based detection is assessment of other cell viability indicators using fluorescent dyes, including membrane potential and membrane integrity (Berney et al., 2007; Stiefel et al., 2015). The LIVE/DEAD^®^ BacLight^TM^ Bacterial Viability Kit (BacLight Kit) differentiates live and dead cells using membrane integrity as a proxy for cell viability and is based on a dual staining procedure using SYTO 9 and propidium iodide (PI) (Berney et al., 2007; Stiefel et al., 2015). The excitation/emission maxima of these dyes are ∼480/500 nm for SYTO 9 and ∼490/635 nm for PI (Invitrogen, 2004). Both dyes intercalate with nucleic acids resulting in an enhanced fluorescent signal; however, they differ in their membrane permeability properties (Freire et al., 2015; Stiefel et al., 2015). SYTO 9 can cross all bacterial cell membranes facilitating a whole cell count when used alone while PI can only enter cells with disrupted membranes allowing differentiation between live and dead cells based on the relative green and red fluorescence from SYTO 9 and PI staining (Berney et al., 2007; Freire et al., 2015; Stiefel et al., 2015). Previous work has demonstrated that SYTO 9 and PI staining of live and dead cell suspensions of *Escherichia coli* can yield a reliable absolute cell count (down to 2.5% live and 20% dead) when using a flow cytometer and reference beads (Ou et al., 2017).

Fluorescence measures are typically taken using a microscope, fluorometer, or flow cytometer (Ambriz-Aviña et al., 2014). Fluorometers and flow cytometers, including top of the range models, have limited spectral resolution while the Optrode allows for the rapid collection of high resolution spectra. The additional information available in high resolution spectra permits detection of subtle differences in spectra and allows for a more refined spectral analysis. At present, the absolute quantification of live and dead cells in a sample can only be done effectively using flow cytometry (McClain et al., 2001). The disadvantages of using flow cytometers are cost and portability (Ambriz-Aviña et al., 2014). Advances in portability have been made in recent years, such as the Accuri C6 from BD Biosciences; however, applicability of flow cytometers is still limited by cost. A cheaper alternative to using a flow cytometer for quantitative detection of fluorescence is a time-resolved fibre-based spectroscopic system called the Optrode developed at the University of Auckland (Hewitt et al., 2012). In brief, the Optrode functions by guiding a laser through a fibre probe into a sample, which in turn collects emitted light from the sample and directs it to a spectrometer (Vanholsbeeck et al., 2014). The spectrometer records the fluorescence signal, and is connected to a computer for further spectral analysis (Vanholsbeeck et al., 2014). Fluorescence based detection of bacterial cells has been achieved using the Optrode for *E. coli* stained with acridine orange (Guo et al., 2017).

Detection of cell viability using the BacLight Kit has several advantages when compared to colony counting on agar plates. Live/dead staining is rapid, and cell death for any given process can be quantified directly and in near real time, rather than retrospectively, from knowing both the input cell number and colonies formed (Berney et al., 2007; Stiefel et al., 2015). The method permits detection of cell states other than live, as those able to grow on an agar plate, and dead, as those unable to grow, including live injured cells that are unable to grow on agar plates (Davis, 2014; Stiefel et al., 2015; Léonard et al., 2016). The fastidious nature of bacteria means that culture-based assessment of viability can result in an underestimation of viable cells, which can impact potential applications (Suller and Lloyd, 1999; Davis, 2014; Léonard et al., 2016). Finally, live/dead staining allows the loss of membrane integrity to be visualised over time (Nocker et al., 2017).

Application of the BacLight Kit for assessing viability of bacteria has been well studied. A variety of dye concentrations, dye incubation times, bacterial strains, growth and staining media, and excitation and emission wavelengths have been used with mixed results (Prakash Singh, 2006; Berney et al., 2007; Hoerr et al., 2007; Kort et al., 2010; Thomas et al., 2011; Eberhart et al., 2012; Freire et al., 2015; Stiefel et al., 2015; Park and Kim, 2018). A majority of the studies have followed the BacLight Kit instructions – growth of cells in rich media and washing in saline before staining, establishment of live and dead cell suspensions to generate a standard curve, and calculation of the red to green fluorescence ratio to determine the proportion of live cells in a sample (Prakash Singh, 2006; Hoerr et al., 2007; Eberhart et al., 2012; Freire et al., 2015; Stiefel et al., 2015; Park and Kim, 2018). However, none of the published studies have established an assay in which cultures are grown and stained in the same media, and no improvements on the green to red fluorescence ratio have been made. Furthermore, the differences between staining with each dye separately and staining in combination have not been explored except in one study (Armstrong and He, 2001). Several studies have applied fluorescence-based methods to AST; however, these methods were complex, employing a range of dyes to assess various viability indicators with promising but mixed results (Walberg et al., 1996; Suller and Lloyd, 1999; Gauthier et al., 2002). Therefore, there is potential for application and optimisation of the use of the BacLight Kit for AST.

In this work we investigate experimental parameters with the aim of developing an improved AST assay to detect antibiotic activity on *E. coli* MG1655 using the Optrode and BacLight Kit as a measure of cell viability. We show that the BacLight Kit can be used to detect live and dead *E. coli* MG1655 suspensions in both saline and minimal A salts medium with 0.2% glucose. These data were used to optimise a model for SYTO 9 and PI fluorescence intensity relative to a live and dead population. Comparison between the results in saline and minimal media permitted validation of using the latter medium for live/dead spectrometry with *E. coli* MG1655. One notable advantage of using minimal media is growth and staining of bacteria occurring in the same media, negating the need to wash samples. A more sophisticated formula for calculating the proportion of live cells in a sample, the adjusted dye ratio, was demonstrated using our model to be superior to the green to red fluorescence ratio that is recommended in the BacLight Kit instructions. During the course of this work we investigated the interaction between SYTO 9 and PI, concluding that the interaction changes from additive to excitation of PI by SYTO 9 as dead cell concentrations increase.

## Materials and Methods

### Bacterial Strain

*Escherichia coli* K-12 strain MG1655 (referred to as *E. coli* MG1655) was selected for live/dead detection in this work as it is a well characterised and widely used strain (Sezonov et al., 2007). *E. coli* MG1655 was grown at 37°C, with shaking at 200 rpm where appropriate.

### Media and Chemicals

Cell biology reagents were purchased from Sigma-Aldrich. General reagents and chemicals included sodium chloride, tryptic soy broth (TSB), agar, and isopropanol. *E. coli* was cultured in TSB or in minimal A salts medium. Minimal A medium was used to support growth in a minimal environment providing only essential nutrients. One litre of a 5× minimal A solution was made according to the following recipe: 5 g (NH_4_)_2_SO_4_, 22.5 g KH_2_PO_4_, 52.5 g K_2_HPO_4_, 2.5 g HOC(COONa)(CH_2_COONa)_2_⋅2H_2_O (sodium citrate⋅2H_2_O). After autoclaving, this solution was diluted to 1× with sterile water and the following sterile solutions, per litre: 1 ml of 1 M MgSO_4_⋅7H_2_O and 10 ml of a 20% w/v glucose solution. The LIVE/DEAD^®^ BacLight^TM^ Bacterial Viability Kit (L7012) was purchased from Invitrogen.

### Preparation of Live and Dead *E. coli* Cultures

Live and dead *E. coli* cultures were prepared based on the instructions given with the BacLight Kit (Invitrogen, 2004). For experiments conducted in saline the following steps were taken: a turbid overnight culture of *E. coli* in TSB was subcultured at a 1:20 dilution in fresh TSB and grown for about 30 min until an OD_600_ of 0.4–0.6 was reached. The subcultured cells were harvested by centrifugation at 7,000 × *g* for 7 min at room temperature and concentrated 10-fold in 0.85% (w/v) saline. For live cells the subculture was diluted 10-fold in saline. For dead cells the subculture was diluted 10-fold in 70% isopropanol. Live cell and dead cell suspensions were incubated at 28°C with 200 rpm shaking for 15 min. The live and dead cells were harvested by centrifugation at 7,000 × *g* for 7 min at room temperature and the pellets were resuspended in saline at approximately 1 × 10^8^ CFU/ml (an OD_600_ of 0.255). Live cells were retrospectively enumerated using the spread plate method. Dead cell numbers were derived from the live cell plate counts. Spread plates from the dead cell suspension were used to confirm cell death had occurred. Experiments conducted in minimal media used the same protocol except the subculture was grown for 3.5 h reflecting the slower growth rate in minimal media. For experiments concerning protocol optimisation, 1 × 10^8^ CFU/ml live and dead suspensions of *E. coli* were mixed to create suspensions containing 0, 1, 2.5, 5, 10, 25, 50, 75, 90, 95, 97.5, 99, and 100% live (ranging between ∼1 × 10^6^ – 1 × 10^8^ CFU/ml). For experiments focussed on investigation of dye interactions, suspensions were prepared at 0, 25, 50, 75, and 100% live.

### Staining Samples With SYTO 9 and PI

Working solutions of SYTO 9 and PI were established in 0.85% saline in amber microcentrifuge tubes (SSIbio) at 33.4 and 400 μM, respectively, and were stored on ice. Stained samples were established by adding 50 μl SYTO 9 working solution, 50 μl PI working solution and/or 0.85% saline (for a total volume of 100 μl) to 0.9 ml culture in an amber tube. Final concentrations of SYTO 9 and PI were 1.67 and 20 μM. For optimisation experiments in minimal media, each live and dead cell suspension was stained with SYTO 9, PI, and SYTO 9 with PI. For optimisation experiments in saline and the investigation of dye interaction, each live and dead cell suspension was stained with PI alone and SYTO 9 with PI. Samples were mixed on a vortex platform for 15 min.

### Treatment of *E. coli* With SYTO 9 and PI to Determine the Impact on Viability

A 1 × 10^8^ CFU/ml subculture was prepared as stated above and aliquoted into four tubes – for SYTO 9 treatment, PI treatment, SYTO 9 and PI treatment, and an untreated control. The samples were incubated at 37°C with 200 rpm shaking. At each time point – 0.25, 0.5, 2, and 5 h – aliquots were taken from all treatments for enumeration by plate counts and fluorescence intensity measured on the Optrode. For treated samples, a 1 ml aliquot was added to an amber tube for Optrode measurements. For the untreated control, a 0.9 ml aliquot was stained with SYTO 9, PI, and SYTO 9 with PI, as described above.

### Measuring Sample Fluorescence With the Optrode

The Optrode, a fluorometer, was used to excite the samples then collect and measure the light emitted as a result of the excitation. Measurements were taken using a continuous measurement script that synchronises the laser and the spectrometer to take 20 ms integration time measurements continuously for a total time of 10 s. Three measurements were taken from each tube and the probe was washed in 70% ethanol between readings. Background readings were taken in triplicate from unstained media except for the investigation of growth media fluorescence for which unstained saline was used for background readings. The power of the laser, kept around 10 mW, was monitored during the measurement using a power meter and photodiode for each experiment.

### Processing of Optrode Data Files

A script in R was used to process the data files (hdf5) generated by the Optrode measurements (R Core Team, 2013). This script sums the first three collected spectra (effective integration time 60 ms), normalises the sum to 1 ms and 1 mW and then subtracts the average background spectrum normalised to the same conditions. The user can define wavelength ranges to be integrated and the number of consecutive spectra to be used for analysis. The output is a data table containing normalised fluorescence intensities (given as relative fluorescence units, RFU) for the wavelength ranges for each measurement taken.

For the optimisation experiments, the fluorescence intensities across the following spectral ranges were investigated: 505–515 nm, 509–529 nm, and 510–540 nm for SYTO 9 emissions, and 600–610 nm, 609–629 nm, and 620–650 nm for PI emissions. The BacLight Kit instructions recommend using 510–540 nm and 620–650 nm. The other wavelength integration ranges were picked based on previous work that identified these ranges as having more characteristic SYTO 9 and PI peaks than the ranges outlined in the BacLight Kit instructions (Ou et al., 2016). For the investigation of dye interaction, the fluorescence intensities of the measured samples across the 600–610 nm wavelength range was examined.

### Biological Data Processing and Statistical Analysis

The fluorescence intensities of SYTO 9 and PI derived from integration at defined wavelength ranges can be used to generate the proportion of live cells in a sample using the formula given in the BacLight Kit instructions, referred to as the kit ratio, defined in equation (1).

(1) $$proportion\;of\;live\;cells\propto \;\frac{SYTO\;9}{PI}$$

Where *SYTO 9* and *PI* are the fluorescence intensities integrated over a defined wavelength range for SYTO 9 and PI, respectively.

However, previous work on this project observed that the relationship between the kit ratio and percentage of live bacteria was not linear (Ou et al., 2016). Therefore, a more sophisticated relationship was developed, called the adjusted dye ratio, equation (2) (Ou et al., 2016).

(2) $$\%\;live\;cells\propto \left(100\times \frac{SYTO\;9}{PI}\right)/\left(1+\frac{SYTO\;9}{PI}\right)$$

For the optimisation experiments, SYTO 9 and PI emissions at defined wavelength ranges were used to generate scatter plots of fluorescence intensity vs. the experimentally determined % live for each staining condition. For the investigation of dye interaction, fluorescence intensity at 600–610 nm was plotted against the experimentally determined % live. For data obtained from the optimisation experiments, the proportion of live cells, as derived from the kit formula and the adjusted dye ratio, was used to generate a scatter plot against the experimentally determined % live.

Each scatter plot was analysed using a linear regression analysis, which fits a straight line to generate the best-fit value of the slope and intercept. The goodness of fit of the line is quantified by the coefficient of determination (*R*^2^), which is a fraction between 0.0 and 1.0. Higher values indicate that the model fits the data better, i.e., the closer an *R*^2^-value is to 1, the greater the fraction of the total variance of Y is explained by the model. The *R*^2^-value was used to inform which wavelength range is associated with the best modelling of % live. Statistical analysis was performed using GraphPad Prism software version 7 (GraphPad Software, Inc.).

For the investigation of the effect of SYTO 9 and PI on cell viability over time, significant difference between conditions was determined by calculating the area under the curve (AUC) for each treatment and comparing the AUC values using a Kruskal-Wallis test with a *P*-value of <0.05 indicating an overall significant difference. A Dunn’s multiple comparison *post hoc* test was then used to look for significant differences between the treatments with a *P*-value of <0.05 indicating a significant difference.

For the investigation of dye interaction, significant difference between conditions was determined using an analysis of covariance (ANCOVA). The ANCOVA first compares the slopes of the lines with a *P*-value of <0.05 indicating a significant difference. If the *P*-value is >0.05 then the ANCOVA compares the intercepts of the lines with a *P*-value of <0.05 indicating a significant difference. The normalised absolute difference in fluorescence intensity between *E. coli* stained with PI alone and PI with SYTO 9 was calculated using equation (3). This data was analysed using a Kruskal-Wallis test with a *P*-value of <0.05 indicating an overall significant difference. A Dunn’s multiple comparison *post hoc* test was then used to look for significant differences between the fluorescence intensity from each live and dead cell suspension with a *P*-value of <0.05 indicating a significant difference.

(3) Normalised difference in red emissions=(SYTO 9  & PI-PI)/PI

Where *SYTO 9* & *PI* and *PI* are the red fluorescence intensities integrated at 600–610 nm for SYTO 9 & PI and PI only, respectively.

## Results

### Investigation of Media

**FIGURE 1:**
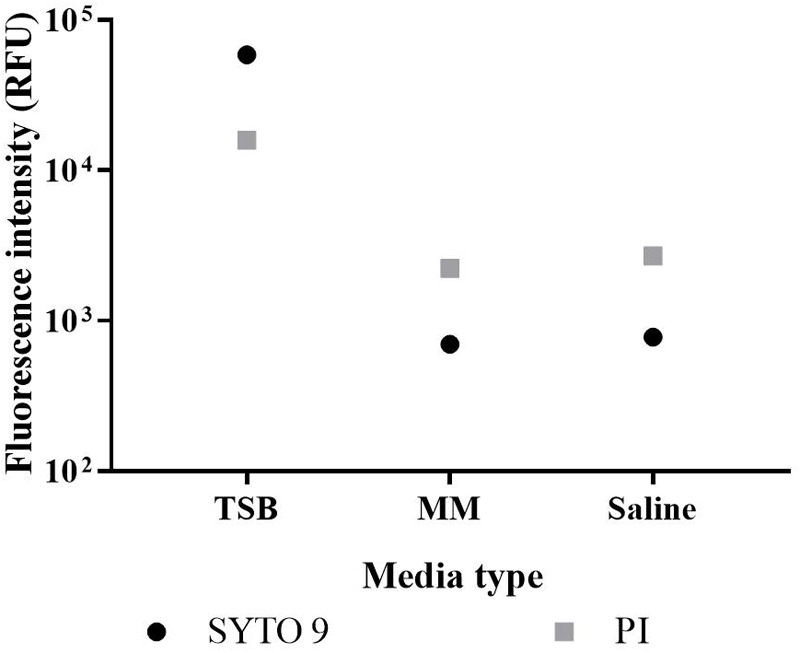
Fluorescence of growth media. TSB, minimal A salts medium with 0.2% glucose (MM), and 0.85% saline (control) were stained with SYTO 9 or PI. Fluorescence intensity was derived from integrating spectra at 505–515 nm for SYTO 9 emissions (circles) and 600–610 nm for PI emissions (squares) with the median value of triplicate readings presented.

Rich media (TSB) and minimal media (minimal A salts with 0.2% glucose) were compared to saline, which does not support growth necessary for AST. The fluorescence signals of SYTO 9 and PI in saline and minimal media were similar, while in TSB intensities were almost two orders of magnitude stronger (Figure 1). The rich media is not appropriate to use in a non-washing protocol. The minimal medium was selected for further testing.

### Optimisation of Data Analysis

**FIGURE 2:**
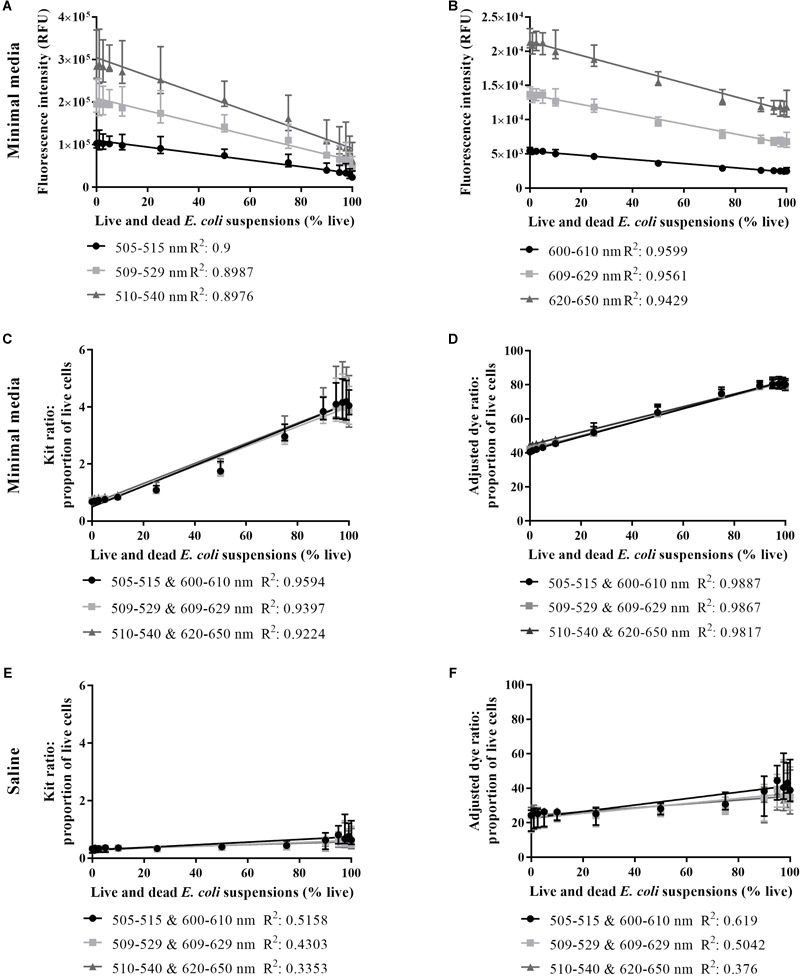
Live/dead spectrometry on live and dead *E. coli* suspensions in minimal media and saline. SYTO 9 intensity in minimal media **(A)**, PI intensity in minimal media **(B)**, proportion of live cells in minimal media derived from the kit ratio **(C)**, % live cells in minimal media derived from the adjusted dye ratio **(D)**, the proportion of live cells in saline derived from the kit ratio **(E)** and % live cells in saline derived from the adjusted dye ratio **(F)** for live and dead *E. coli* suspensions derived from integrating at three different wavelength ranges for each dye. An *R*^2^-value was generated from a linear regression analysis of data from each wavelength range. Data presented is the median with the range from six biological replicates.

Several parameters beyond those outlined in the BacLight Kit instructions were investigated. Integration across the wavelength ranges 505–515 nm and 600–610 nm for SYTO 9 and PI, respectively, resulted in the most linear fits when comparing signal intensity or dye ratio to the proportion of live cells in the *E. coli* suspension, in minimal media and saline (Figure 2A–F). In comparison, lower *R*^2^-values were obtained with 509–529 nm and 510–540 nm for SYTO 9, and 609–629 nm and 620–650 nm for PI (Figure 2A,B). Similarly, lower *R*^2^-values were obtained with 509–529 nm and 609–629 nm, and 510–540 nm and 609–629, for the proportion of live cells calculated from the kit and adjusted dye ratio (Figure 2C,D).

The feasibility of using the kit or adjusted dye ratio for ascertaining the proportion of live cells in a stained culture was examined in minimal media and saline using 505–515 nm and 600–610 nm wavelength ranges. In minimal media and saline, the smaller error bars and the higher *R*^2^-value for the adjusted dye ratio compared to the kit ratio supported the use of the adjusted dye ratio (Figure 2C–F). The relationship between signal intensity or adjusted dye ratio was strongest when minimal media was used (Figure 2D, F). These results further support the detection of live and dead cells using SYTO 9 and PI in minimal media.

### Limit of Detection

**FIGURE 3:**
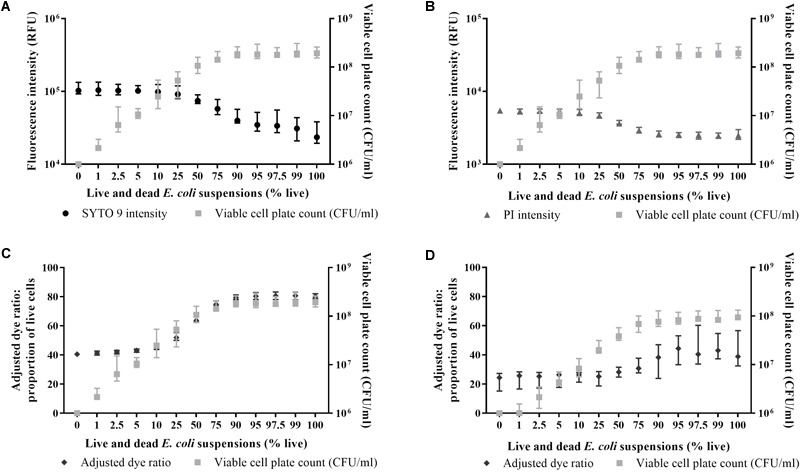
Relationship between live/dead spectrometry and viable cell plate counts in minimal media and saline. SYTO 9 emissions in minimal media (**A**; circles), PI emissions in minimal media (**B**; triangles), the adjusted dye ratio in minimal media (**C**; diamonds), and the adjusted dye ratio in saline (**D**; diamonds) compared to viable cell plate counts (**A–D**; squares) for live and dead *E. coli* suspensions. Fluorescence intensity was obtained from integrating 505–515 nm and 600–610 nm. Data presented is the median with the range from six biological replicates.

In minimal media SYTO 9 and PI emissions decrease as % live increases (Figure 3A,B). The % live calculated from the adjusted dye ratio decreases in accordance with the experimentally determined % live until the former plateaus at ∼5% live (∼5 × 10^6^ CFU/ml) (Figure 3C). In saline, the % live calculated from the adjusted dye ratio decreases as the experimentally determined % live decreases until ∼25% live (∼2.5 × 10^7^ CFU/ml), after which it plateaus (Figure 3D).

### The Effect of SYTO 9 and PI Incubation on *E. coli* Viability

**FIGURE 4:**
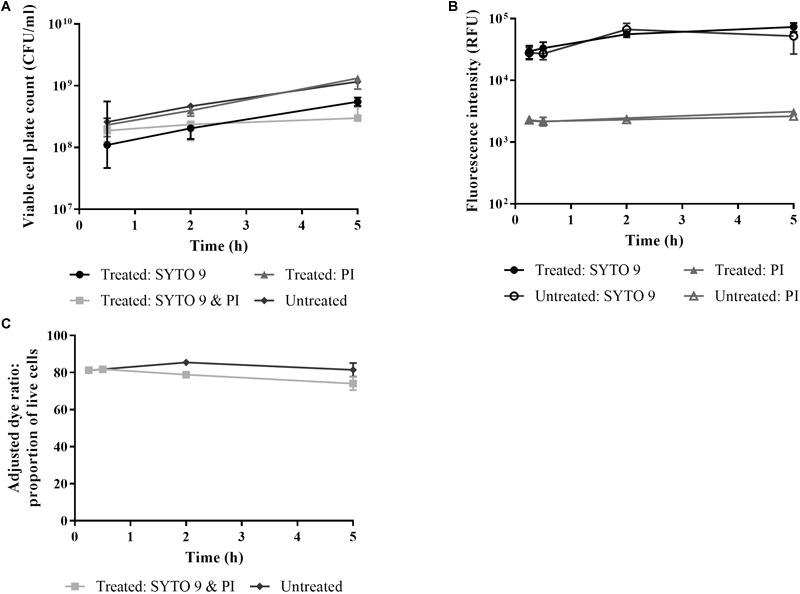
The effect of live/dead staining on *E. coli* viability. *E. coli* cells in minimal media were stained with SYTO 9 and PI individually and in combination over time. Untreated cells were stained at each time point. At 0.25, 0.5, 2, and 5 h time points aliquots were taken for determination of cell viability by viable cell plate counts **(A)** and by measuring fluorescence of stained cultures **(B,C)**. Fluorescence intensity was obtained from integrating 505–515 nm (circles) and 600–610 nm (triangles). The adjusted dye ratio (squares) was used to determine the % live. Data presented is the median with the range from three biological replicates.

The effect of staining the sample at the start of a time course experiment, thereby allowing sample fluorescence to be measured immediately, was investigated (Pore, 1994). After a 5 h treatment, incubation with both dyes has the greatest inhibitory effect (∼0.5–1 log reduction) on cell numbers followed by SYTO 9 (∼0.5 log) as defined by viable plate counts (Figure 4A). *E. coli* cells incubated with PI were present at similar viable cell plate counts to untreated cells (Figure 4A). Higher fluorescence intensities were recorded from cells incubated with SYTO 9 and PI individually compared to untreated cells (Figure 4B), possibly indicative of a reduction in viability as higher fluorescence intensities were detected for isopropanol killed cells compared to untreated cells (Figure 3A,B). The adjusted dye ratio of samples incubated with both dyes was lower than the untreated control (Figure 4C), suggesting that incubation with the dyes lowers cell viability (Figure 4A).

### Investigation of Dye Interaction

**FIGURE 5:**
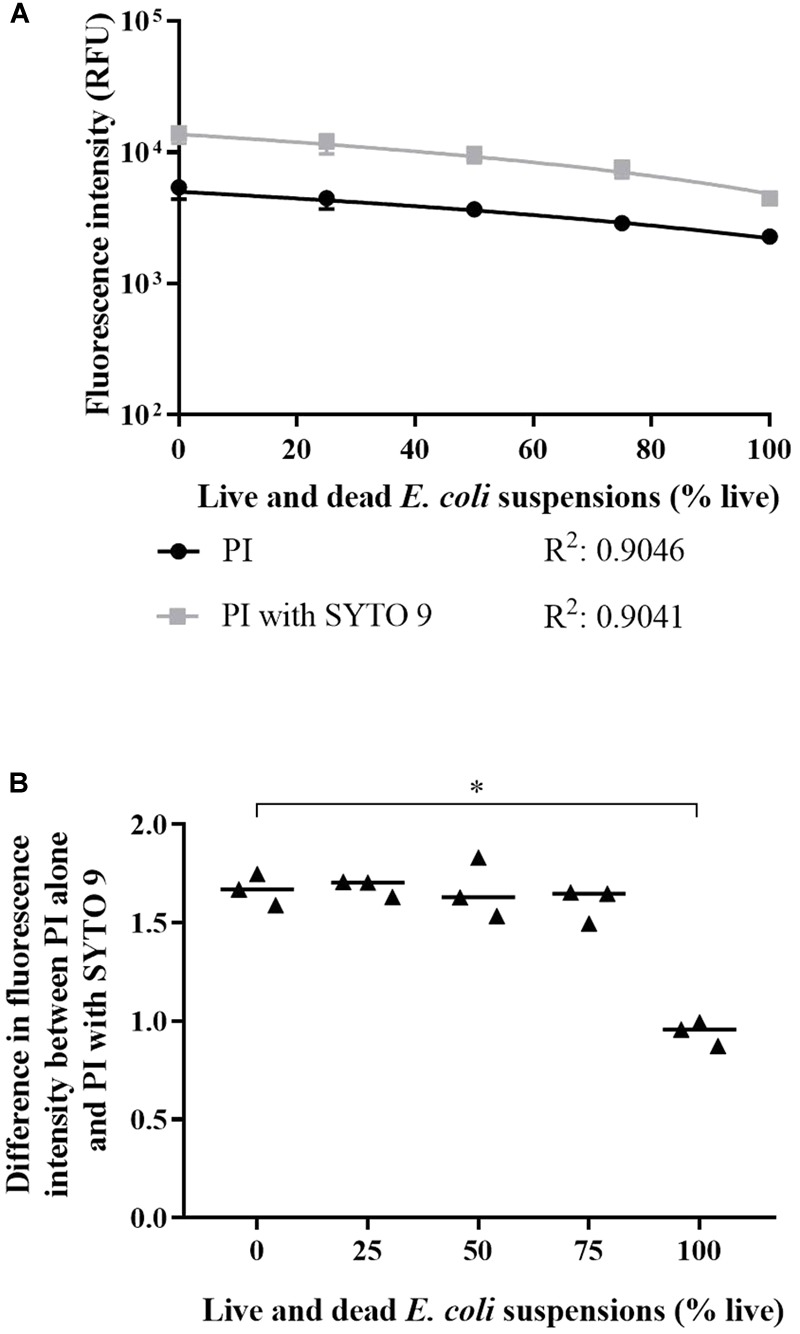
Interaction between SYTO 9 and PI. **(A)** Fluorescence intensities at 600–610 nm from live and dead *E. coli* suspensions in minimal media stained with PI alone (circles) and PI with SYTO 9 (squares). A line of best fit for each staining condition was generated using a linear regression analysis and significant difference between conditions was determined using an ANCOVA. The slopes of the lines were significantly different (*P*-value: <0.05). Data presented is the median with the range from three biological replicates. **(B)** The absolute difference in fluorescence intensity between *E. coli* stained with PI alone and PI with SYTO 9 normalised to PI alone. Statistical significance is represented by ^∗^ (Kruskal-Wallis test, *P*-value: <0.05, Dunn’s multiple comparison *post hoc* test). Data presented is from three biological replicates with a median line.

The fluorescence intensity (600–610 nm) from *E. coli* stained with SYTO 9 and PI is greater than with PI alone; the presence of SYTO 9 may mediate an increase in red emissions (Figure 5). The enhanced red emissions could be due to spectral overlap or crosstalk, or could be indicative of SYTO 9 emissions exciting PI (Stiefel et al., 2015). Crosstalk occurs when the emissions from one dye overlap the wavelength emission range for a second dye (Armstrong and He, 2001; Stiefel et al., 2015). SYTO 9 emits fluorescence up to ∼650 nm (Armstrong and He, 2001) and therefore can contribute to emissions in the 600–610 nm range, which is usually attributed to only PI. SYTO 9 emissions can excite PI as the emission spectrum of SYTO 9 overlaps with the excitation spectrum of PI (Stocks, 2004), which results in quenching of the green (505–515 nm) emissions and enhancement of the red (600–610 nm) emissions.

Red emissions from cells stained with PI alone and with both dyes were compared. PI fluorescence intensity was significantly greater in the presence of SYTO 9 (Figure 5A; linear regression analysis; slopes are different; *P-*value: <0.05). The absolute intensity difference normalised to the intensity of PI alone was calculated (Figure 5B). The difference in PI intensity increased with dead cell concentration. The difference between 0 and 100% live was statistically significant (Kruskal-Wallis test, *P*-value: <0.05, Dunn’s multiple comparison *post hoc* test).

**FIGURE 6:**
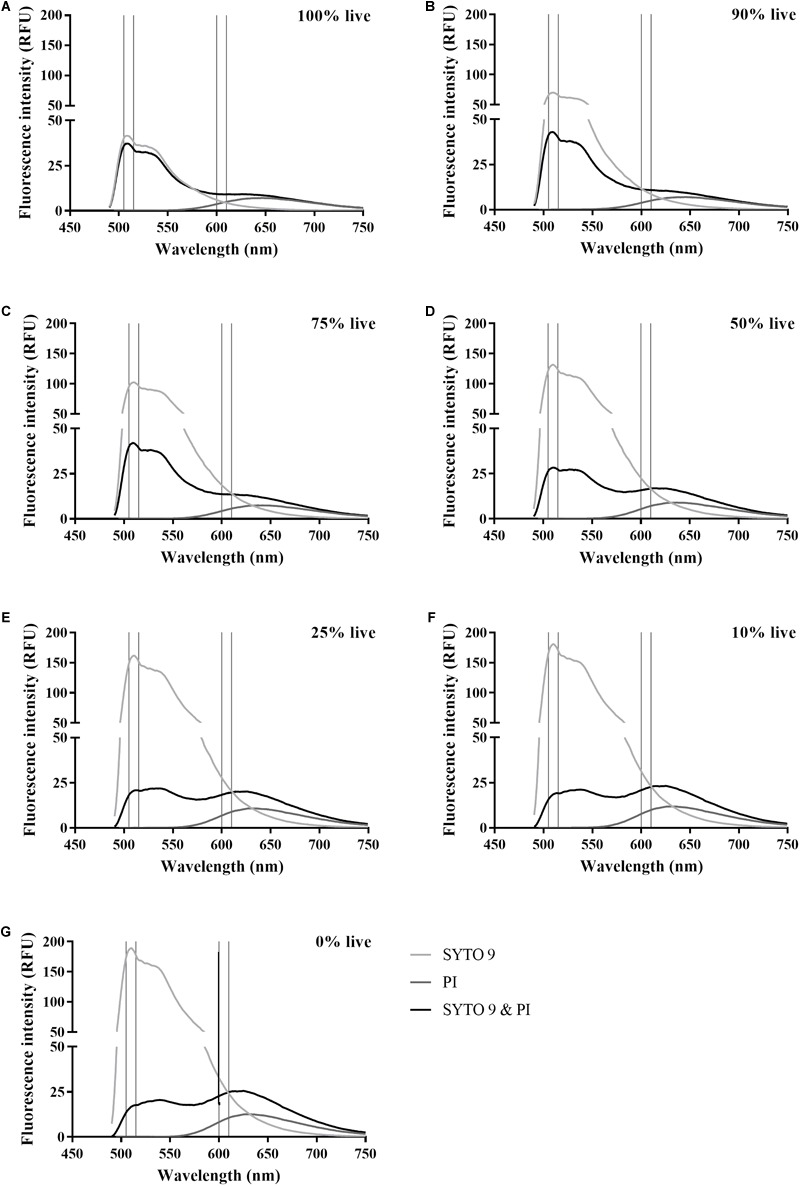
Spectra of SYTO 9 and PI stained live and dead *E. coli* suspensions. Fluorescence spectra of SYTO 9 stained (light grey), PI stained (dark grey), and SYTO 9 and PI stained (black) live and dead *E. coli* suspensions in minimal media. The suspensions are expressed as % live with 100% **(A)**, 90% **(B)**, 75% **(C)**, 50% **(D)**, 25% **(E)**, 10% **(F)**, and 0% **(G)**. Vertical lines indicate the selected wavelength integration ranges at 505–515 nm for SYTO 9 emissions and 600–610 nm PI emissions. The axes of the graphs have been chosen to highlight the interaction of the dyes in the red emission range. Data presented is the median from six biological replicates.

The dye interaction between SYTO 9 and PI was examined in suspensions of a range of live:dead ratios of stained *E. coli* across the 450–750 nm fluorescence spectra (Figure 6). The SYTO 9 emission is much greater than the PI emission, particularly for suspensions with a high concentration of dead cells. For 0–75% live cell suspensions, the emission intensities at 600–610 nm from SYTO 9 are greater than from SYTO 9 with PI while the opposite is true for PI (Figure 6), which supports the assertion that the dyes interact resulting in SYTO 9 signal quenching and PI signal enhancement. However, for 90–100% live cell suspensions, the SYTO 9 emissions (600–610 nm) are less than from SYTO 9 with PI (Figure 6), which suggests that for SYTO 9 and PI stained cells, the signal at this wavelength range is attributed to overlap of SYTO 9 emissions combined with PI unbound dye emissions. A dead cell concentration threshold exists between the 75 and 90% live cell suspensions, which we hypothesise marks the point where there is a sufficiently high dead cell concentration to facilitate binding of both dyes to DNA and the subsequent quenching/enhancement interaction.

## Discussion

We have adapted the protocol outlined in the BacLight Kit instructions to apply live/dead spectrometry to *E. coli* populations containing live and dead cells. The optimised protocol can be potentially applied to AST as an alternative to culture-based methods. First an appropriate medium was selected. The BacLight Kit instructions recommend a rich medium for growing test organisms and 0.85% saline for staining. To reduce the number of steps involved in live/dead spectrometry, a single medium suitable for both growing and staining the bacteria is required. TSB, a rich media, contains components that fluoresce following staining with SYTO 9 and PI, particularly for the former, while the minimal medium yielded similar fluorescence intensity as saline (Figure 1). Fluorescence intensity from unwashed samples in minimal A salts medium can be measured, which prevents loss of biological information from the samples and reduces experimental error (Ou et al., 2017).

Most research conducted using the BacLight Kit follow (Eberhart et al., 2012; Kahlisch et al., 2012; Stiefel et al., 2015) the staining instructions, sometimes with slight modifications, including staining in buffer (Leuko et al., 2004; Jung et al., 2008; Freire et al., 2015) and water (Hoefel et al., 2003) for viability detection of planktonic bacterial cells. These media do not support reasonable bacterial growth and are therefore not suitable for AST. One study has attempted to overcome the problem of needing to wash samples of antimicrobial challenged bacteria by using a rich media for initial growth then challenging *E. coli* in a non-fluorescent media containing saline and glucose (Anaya et al., 2016). However, the glucose (0.194–0.777 mM) concentrations used are low compared to minimal A salts medium (11.1 mM), and would not support growth sufficiently for reliable AST (Anaya et al., 2016). The staining methodology presented in the current report fills a gap in the literature pertaining to AST using fluorescence-based measures of cell viability.

We determined the optimal wavelength integration ranges and formula to calculate the proportion of live cells (Figure 2). The experimental parameters outlined in the BacLight Kit protocol – staining in saline, wavelength integration ranges (510–540 nm and 620–650 nm), and the kit ratio – generated data with a poor fit. In contrast, staining in minimal media, 505–515 nm and 600–610 nm wavelength integration ranges, and the adjusted dye ratio generated data with an improved fit. Our adapted experimental model of growing and staining *E. coli* MG1655 cells in minimal media allows for adequate determination of the proportion of live cells and is an improvement on the BacLight Kit instructions.

The better fit obtained in minimal media compared to saline may be indicative of differences in cell staining. For SYTO 9 and PI stained 100% to 0% live suspensions, the 505–515 nm emissions from cells ranged 1.8 × 10^3^–4.01 × 10^3^ relative fluorescence units (RFU) and 2.11 × 10^4^–9.28 × 10^3^ RFU, in saline and minimal media, respectively. The 600–610 nm emissions from cells ranged 2.68 × 10^3^–1.26 × 10^4^ RFU and 5.36 × 10^3^–1.33 × 10^4^ RFU in saline and minimal media, respectively. The higher fluorescence intensities demonstrate that the *E. coli* cells in minimal media took up a greater amount of SYTO 9 and PI. Therefore, staining in minimal media has greater sensitivity to cell concentration than staining in saline.

The improved staining of *E. coli* cells in minimal media may reflect a more active state. It has been previously observed that *E. coli* in log phase had higher fluorescence intensity compared to stationary phase when stained with SYBR green and PI (Barbesti et al., 2000). More active cells will have a higher RNA content increasing the number of binding sites for the dyes while nutrient scarcity can cause cell wall alterations reducing dye permeability (Berney et al., 2007). The enhancement of staining in minimal media is greater for 100% live cell suspensions (∼1 log) compared to 0% live cell suspensions (∼0.5 log), which supports the assertion that signal enhancement is due to active live cells.

For live/dead spectrometry, a media and strain specific calibration curve is required (Figure 2C; Freire et al., 2015). The calibration curve can inform about the limit of detection (LoD) and can be used to confirm the suitability of the adjusted dye ratio to calculate the proportion of live cells in a sample (Freire et al., 2015; Ou et al., 2017). Detection of live cells in live and dead cell suspensions plateaued at ∼5% live in minimal media (Figure 3C) and 25% live in saline (Figure 3D). The lower LoD further validates the application of live/dead spectrometry to *E. coli* in minimal media. Previous work examining live/dead staining of *E. coli* 25922 using a flow cytometer demonstrated a LoD down to 2.5% live and 20% dead bacteria in live and dead suspensions (Ou et al., 2017). No comment can be made on the LoD of dead cells using the current optimised methodology as enumeration by plate counts only informs about dead cell counts indirectly. Future work will further characterise the LoD using a flow cytometer. While the LoD for live cells does not extend to a 99.9% reduction in viable cell plate counts, as required for determination of bactericidal activity, the results demonstrate the feasibility of quantifying differences in the viability of *E. coli* populations using the optimised methodology. Future work will focus on measurement of single cell fluorescence spectra to identify unique spectra signatures for viability states, and thus not be limited by the current LoD. Single cell measurements can be achieved using microfluidic chips – small platforms capable of separating and concentrating cells for subsequent imaging (Kaminski et al., 2016). Microfluidics is a promising next step as it is associated with minimal consumption of consumables and bacterial samples, and more precise liquid handling (McClain et al., 2001; Kaminski et al., 2016; Lee et al., 2017).

Staining a sample at time zero to negate the requirement for a 15 min staining period at each time point was investigated. We detected an inhibitory effect of the dyes, especially for SYTO 9 and PI together (Figure 4). While the differences were not statistically relevant, the trends are informative as anything that impacts on cell physiology and growth could skew results. Therefore, for reliable live/dead testing, the dyes should be added to aliquots taken at each time point.

The BacLight Kit suggested that PI displaces SYTO 9 resulting in emissions from cells with compromised membranes to shift from green to red. However, this only occurs at certain relative dye and nucleic acid concentrations (Stocks, 2004). In a cell-free system of DNA stained with different proportions of PI and SYTO 9, PI needed to saturate nucleic acid (>0.4 M PI to 1 M DNA base pairs) for displacement to occur (Stocks, 2004). When nucleic acid was in excess of PI there was space for both dyes to bind the DNA (Stocks, 2004). Staining with both dyes resulted in quenched green and enhanced red emissions suggesting that dye interaction was occurring (Stocks, 2004).

We investigated the occurrence of dye interaction. SYTO 9 enhanced PI emissions for all live and dead cell suspensions tested (Figure 5A). The normalised difference in emission intensity at 600–610 nm between PI alone and PI with SYTO 9 (equation 3) showed that ≥25% dead cells are needed for PI notable emission enhancement by SYTO 9 (Figure 5B), which is supported by the spectra presented in Figure 6. This reflects greater amounts of interaction occurring due to greater SYTO 9 and PI staining of dead cells (Figure 3A,B) relative to live cells, resulting in increased opportunities for interaction to occur. Dead *E. coli* cells are thought to have increased SYTO 9 staining relative to live cells because inner and outer membranes of live cells are not completely permeable to SYTO 9 (Stiefel et al., 2015). The impact of dye interaction was only significant for 0% vs. 100% live (Figure 5B) i.e., in dead cells where the maximum amount of PI has bound to DNA and can interact with SYTO 9. The influence of the degree of SYTO 9 and PI staining on dye interaction is further demonstrated by the fluorescence intensity of dual stained live and dead cell suspensions at 505–515 nm. The fluorescence intensities of 100% to 0% live suspensions ranged from 2.11 × 10^4^–9.28 × 10^3^ RFU in minimal media and 1.8 × 10^3^–4.01 × 10^3^ RFU in saline. In minimal media, the decrease in fluorescence intensity as dead cell concentration increases suggests that SYTO 9 emissions are quenched concomitantly with PI emission enhancement. Conversely, in saline the fluorescence intensity increased with dead cell concentration, which may be reflective of a lower degree of SYTO 9 and PI staining and therefore a reduced amount of dye interaction.

We observed enhanced 600–610 nm emissions from cells stained with SYTO 9 and PI compared to PI alone (Figure 5), which is indicative of dye interaction and suggests that PI has not displaced all SYTO 9. The 505–515 nm emissions from 0% live *E. coli* suspensions stained with SYTO 9 and PI supports this conclusion (Figure 6). Emissions at 600–610 nm from 100% live cells stained with SYTO 9 or PI were less than cells stained with both dyes (Figure 6), which suggests that there is little to no quenching/enhancement occurring. The increased 600–610 nm emissions would likely be due to SYTO 9 emissions combined with unbound PI emissions (background). The dye interaction changes from crosstalk to excitation of PI by SYTO 9 between 90% and 75% live cells as indicated by the relative 505–515 nm emissions between SYTO 9 and SYTO 9 with PI (Figure 6). The 75% live cell suspension contains a sufficiently high concentration of dead cells to allow both dyes to bind and the interaction to occur. The green and red emissions are not solely from live and dead cells, respectively, which must be considered when interpreting live/dead spectrometry results. Further investigation is needed to fully understand the impact of the dye interactions in different conditions.

A fluorescence-based live/dead cell enumeration protocol with attributes fit for AST studies was optimised. Fluorescence measurements from samples established according to the optimised protocol can be taken using any equipment that measures fluorescence, including a flow cytometer, spectrofluorometer, microplate reader, and the Optrode. Benefits of the optimised methodology include no requirement for washing of samples before staining to prevent loss of biological information, utilisation of a non-fluorescent media that supports growth of bacteria necessary to detect antibiotic action, higher levels of staining of *E. coli* cells, and generation of live/dead spectrometry data with a better fit than that generated according to the kit instructions. Disadvantages for this methodology include a high LoD (∼5 × 10^6^ CFU/ml) and a dye interaction that is influenced by the number of dead cells, which could be problematic when testing antibiotic challenged samples in which the number of dead cells are unknown.

## Conclusion

The LIVE/DEAD^®^ BacLight^TM^ Bacterial Viability Kit can be applied to *E. coli* MG1655 in minimal A salts medium with 0.2% glucose to detect viability on samples that did not require washing before staining. Optimal wavelength integration ranges for examining fluorescence emissions from SYTO 9 and PI are 505–515 nm and 600–610 nm, respectively. These integrated peaks areas were used in an adjusted dye ratio that better correlated with the proportion of live cells than the kit ratio. Dye interaction occurs in cells with compromised membranes that have been stained with both SYTO 9 and PI.

## Author Contributions

JR conceived, designed, and performed the experiments, analysed the data, prepared the figures and/or tables, authored drafts of the manuscript, and approved the final draft. CM provided computer code for analysis of the data, reviewed drafts of the manuscript, and approved the final draft. FV and SS were involved in project administration, they advised on the experimental protocol and analysis of the results, reviewed drafts of the manuscript, and approved the final draft.

## Conflict of Interest Statement

The authors declare that the research was conducted in the absence of any commercial or financial relationships that could be construed as a potential conflict of interest.
